# Endoscopic transsphenoidal surgery for resection of pituitary macroadenoma: A retrospective study

**DOI:** 10.1371/journal.pone.0255599

**Published:** 2021-08-06

**Authors:** Jiun-Lin Yan, Chen-Nen Chang, Pin-Yuan Chen

**Affiliations:** 1 Department of Neurosurgery, Chang Gung Memorial Hospital, Keelung City, Taiwan; 2 Department of Neurosurgery, Xiamen Chang Gung Hospital, Xiamen, China; Sapienza University of Rome, ITALY

## Abstract

**Background:**

The endoscopic transsphenoidal approach is an efficient minimally invasive procedure for removal of pituitary tumors that can be accomplished through a one-hand or two-hand approach. The one-hand procedure through one nostril is more intuitive for surgeons, but maneuvering the instruments can be restrictive. The two-hand procedure using a one-and-half nostril approach provides more precise manipulation. This study aimed to compare the surgical outcomes of one-hand/mono-nostril and two-hand/one-and-half nostril surgeries for resection of large pituitary tumors by a single neurosurgeon.

**Materials and methods:**

The surgical data of 78 consecutive cases with pituitary macroadenoma (diameter >1 cm) were reviewed retrospectively. Altogether, 30 cases received one-hand/mono-nostril surgery, while 48 cases received two-hand/one-and-half nostril surgery. Postoperative outcomes of the two operations were compared.

**Results:**

The operative time, hospital stay, residual rate of pituitary macroadenoma, visual field, surgical complications, and re-operative rates were slightly improved in the two-hand/one-and-half nostril surgery group compared with that in the one-hand/mono-nostril surgery group (all *p*>0.05). However, postoperative hypopituitarism was less frequent (1/48; 2.0%) with the two-hand/one-and-half nostril approach than with the mono-nostril approach (*p* = 0.004). Similar surgical outcomes were found in all patients with either small or large pituitary tumors, except that the difference in postoperative improvement in visual field change reached statistical significance (*p* = 0.044).

**Conclusion:**

A single-surgeon endoscopic endonasal transsphenoidal surgery with two-hand/one-and-half nostril approach is an effective and safe procedure for removal of large pituitary tumors.

## Introduction

In the past 20 years, neurosurgeons have used the endoscope to perform transsphenoidal surgery for pituitary tumors resection [1–5]. The endoscope is able to provide a panoramic field of view, better illumination, and the ability to look into corners via angled lenses. Thus, the endoscope has become the instrument of choice for transsphenoidal surgery since it was first advocated by Jho and Carrau [1] in a series of 50 patients published in 1997, and later by Nishioka [4], in 2017. Since the introduction of endoscopic transsphenoidal techniques, many variations and improvements have been made [3, 6–9], including the mono-nostril versus the bi-nostril approach and lateralizing the middle turbinate versus performing a middle turbinectomy [3, 5, 10, 11].

Most groups that have promoted a fully endoscopic technique have used and strongly emphasize—a two-surgeon, three- or four-hand approach [11–16]. In the two-nostrils/four hands technique [17], the primary surgeon removes the soft tissue, bone, and tumor and performs the postresection reconstruction, while a second surgeon controls the endoscope and assists with suction or irrigation. Most often, an otorhinolaryngology (ENT) surgeon makes the initial sphenoid exposure, and a neurosurgeon removes the bony structures of the skull base and the lesion while the ENT surgeon operates the endoscope. This two-surgeon approach benefits from the ENT surgeon’s familiarity with nasal anatomy and sinus endoscopy; the surgeons’ ability to discuss cases and anatomic variations; the ability to rapidly readjust the endoscope position; assistance with hemostasis; and greater familiarity with vascularized pedicle flaps needed for large skull-base repairs [18–20]. An alternative, manpower-saving approach is a single-surgeon operation, with a mechanical or pneumatic endoscope holder [21]. This approach does not vary from the two-surgeon method as long as the surgeon has equivalent ability to visualize structures from a variety of angles and to remove tumor. A single-surgeon approach potentially reduces or eliminates some drawbacks of the two-surgeon method and may make endoscopy more accessible to neurosurgeons. The availability of an endoscope holder that rivals the mobility and ease of use of either the operating microscope or a second surgeon had been a limiting factor.

Several reports have documented the safety and utility of fully endoscopic methods of transsphenoidal surgery for pituitary tumors [11, 12, 15, 22]. Potential benefits, in addition to those mentioned above, include diminished soft-tissue manipulation; opportunity for more extensive resection; enhanced visualization of the operative field; and greater patient satisfaction. However, in addition to evaluating the extent of resection, neurologic and endocrinologic outcomes, it is important to evaluate the complications that may result from the type of approach used. To better assess the role of surgical technique on outcomes of endoscopic transsphenoidal pituitary surgery, we present a single neurosurgeon’s experience with the one-hand/mono-nostril or two-hand/one-and-half nostril, anatomy-preserving (without removing the turbinates) operations, assessing volumetric extent of resection, outcomes, and complications.

## Materials and methods

### Study design and patient selection

The protocol for this study was approved by the Institutional Review Board of Chang Gung Memorial Hospital (No. 201901259B0). A total of 78 patients who had pituitary macroadenoma (> 1 cm), and received purely endoscopic transsphenoidal surgery for tumor resection between 2014 and 2019 were included. All operations were performed by a single surgeon. Exclusion criteria were pituitary carcinoma, double tumor in the sellar region, and lack of post-operative magnetic resonance imaging (MRI) scans performed six months after surgery.

Thirty patients received one-hand surgery through the right nostril; another 48 patients received two-hands/one-and-half nostril approach with the assistance of a scope holder (Martin Arm, Karl Storz, Germany). The distribution of surgical dates was similar between the two groups. Clinical characteristics between two groups were compared. Knosp classification for parasellar extension [23] and Hardy suprasellar extension grading [24] were used for evaluation of tumor invasion. The extent of tumor resection was estimated based on findings during postoperative MRI scan obtained 3–6 months after the surgery.

### Patient characteristics

Preoperative data collected were the presence of hemorrhagic components, optic nerve compression, and tumor size. MR images were assessed to determine tumor suprasellar extension; anterior extension (over the planum sphenoidale); posterior extension (growth into the interpeduncular cistern/prepontine area and/or causing compression of the brainstem); suprasellar lateral extension (beyond the intracranial component of the internal carotid artery [ICA]); and Knosp grade. These assessments were based on Hardy-Vezina and Knosp classification systems. Preoperative pituitary endocrine function laboratory data collected were follicle-stimulating hormone; thyroid-stimulating hormone; T3; T4; cortisol; adrenocorticotropic hormone; insulin-like growth factor-1; and prolactin levels.

At 6 months after the surgery, postoperative characteristics assessed were changes in visual acuity; visual fields; endocrine function; need for postoperative radiation; length of stay; and postoperative hypopituitarism. Hypopituitarism was defined as deficient secretion of one or more pituitary hormones because of pituitary or hypothalamic disease, and diagnosis is made by documenting subnormal secretion of these pituitary hormones in defined circumstances [25]. The hormone testing was typically performed and reported according to the methods of Fatemi et al. [26]. Endocrine function was assessed in a multidisciplinary pituitary clinic with provocative or dynamic testing. The presence of postoperative complications, including diabetes insipidus, cranial nerve palsy; ICA damage; cerebrospinal fluid (CSF) leak; headache; epistaxis; sinusitis; visual complications; intracranial hemorrhage; hydrocephalus; meningitis; coma; and death were recorded.

### Tumor volume measurement

Preoperative volumetric analysis was performed on coronal (width diameter) and sagittal (height and length diameters) gadolinium-enhanced T1-weighted images from the study performed closest to the date of the patient’s operation. Postoperative volumetric analysis was performed to calculate the volume of residual tumor on follow-up MRI, typically performed three to six months postoperatively. Extent of resection was defined as the intra-operative extent of resection (subjectively determined by the surgeon) and postoperative extent of resection (MRI Residual).

### Surgical approach

**Fig 1:**
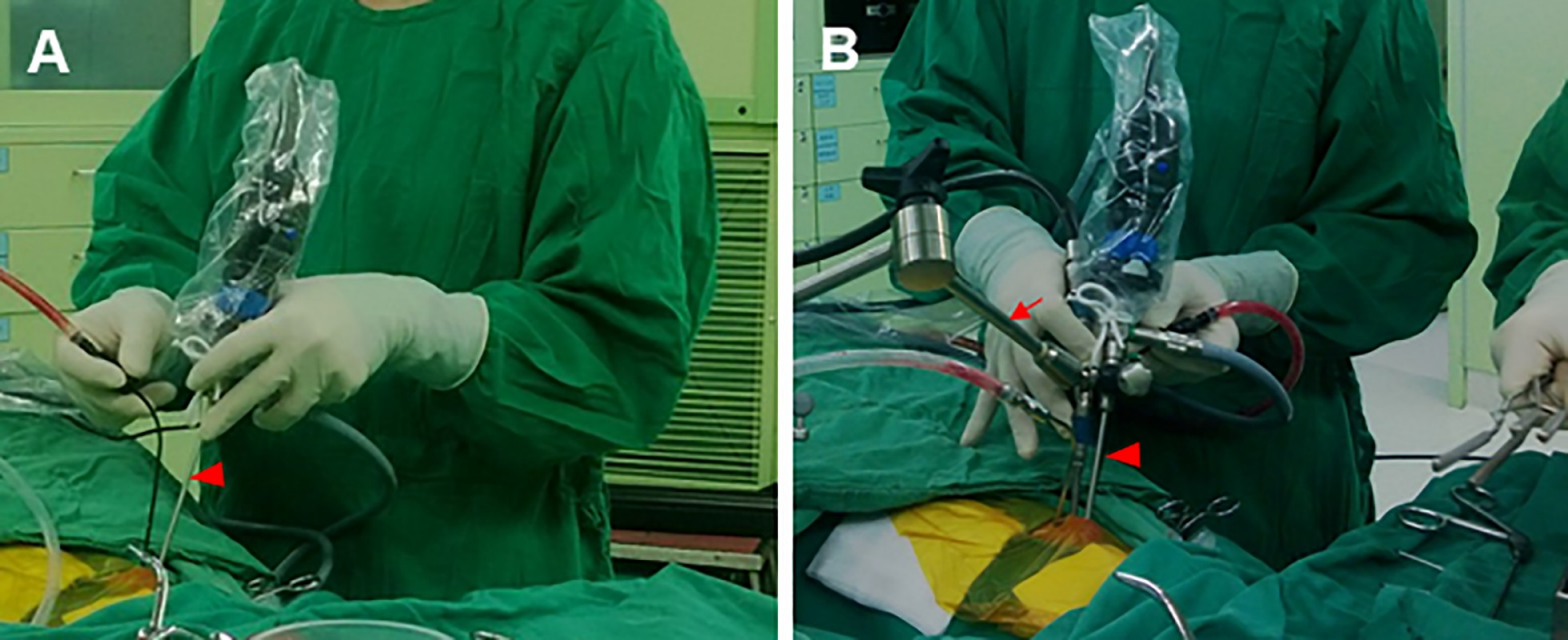
One-hand (A) and two-hand (B) technique surgical views. (A) The one-hand/mono-nostril operation for treatment of pituitary tumors was performed through the right nostril with one hand holding the endoscope (filled triangle) and the other hand using the surgical instruments. (B) The two-hand/one-and-half nostril surgery was assisted by an endoscope holder (arrow). All techniques employed in two-surgeon endoscopic surgery are incorporated into this approach.

All patients underwent transnasal transsphenoidal endoscopic resection of a pituitary adenoma. The objective of surgery was maximum decompression of the optic apparatus—with maximum care taken not to injure sensitive neural and vascular structures, and to preserve or restore endocrine function. The positions of one-hand and two-hand surgery are shown in **Fig 1**.

**Fig 2:**
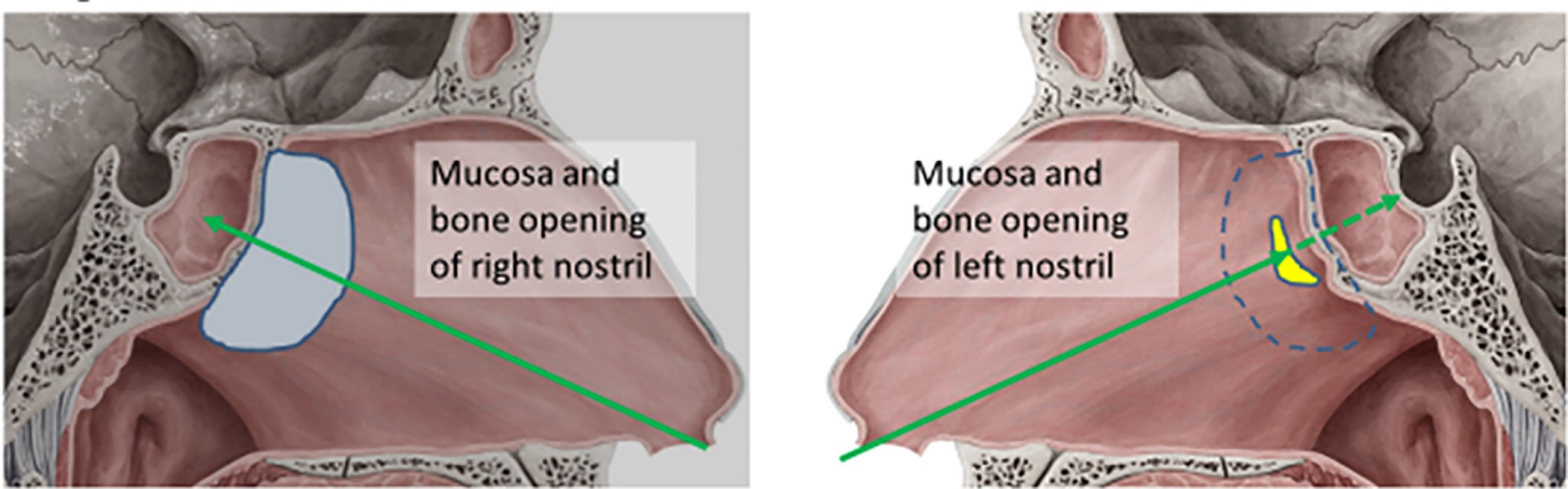
Illustration of surgical approach. (A) The one-hand operation was done through the right nostril with one hand holding the endoscope and the other hand using the surgical instruments. The middle turbinate was pushed laterally to expose the ostium of the sphenoid sinus. A naso-septal flap was created before the sphenoidectomy in selected cases. The sphenoidectomy was performed by widening the ostium with partial removal of the vomer bone. (B) The two-hand/one-and half nostril approach shared a similar surgical approach. After the exposure of the sphenoid sinus, the distal end of the nasal septum was opened for the contralateral nostril approach. The endoscope was then held by the holder, and the opening of the sellar floor and the removal of the tumor was performed by two-hand manipulation. The images were copied from website (https://www.kenhub.com/en/start/medial-wall-of-nasal-cavity) and further modified.

One-hand surgery was performed through the right nostril (**Fig 2A**), with one hand holding the endoscope (Karl Storz, Germany) and the other hand using the surgical instruments. The middle turbinate was pushed laterally to expose the ostium of the sphenoid sinus. A naso-septal flap was created before the sphenoidectomy in selected cases. The sphenoidectomy was performed by widening the ostium with partial removal of the vomer bone. After exposure of the sellar floor, with clear identification of the bilateral ICA groove, the sellar floor was opened with a high-speed diamond burr and removal of the tumor by using ring curettage and suction. Hemostasis was achieved with cotton packing, surgical (oxidized regenerated) cellulose, or FloSeal® (Baxter) when needed. The sellar floor was reconstructed using direct suture and covered with artificial dura mater with or without Tisseel® (Baxter). A naso-septal flap was used in selected cases.

The two-hand/one-and half nostril approach used a similar surgical approach. After exposure of the sphenoid sinus, the distal end of the nasal septum was opened for the contralateral nostril approach (**Fig 2B**). The endoscope was then held by the holder, and the sellar floor was opened and the tumor removed with two-hand manipulation. Reconstruction of the sellar floor was accomplished as with the one-hand operation; the dura was sutured directly in selected cases.

### Statistical analysis

Continuous variables are expressed as mean ± standard deviations (SD), and categorical variables as counts (weighted percentage). Student’s t test was used for normal distribution of continuous variables; Mann-Whitney U test was used for nonparametric analysis. Chi-square test and Fisher’s exact test were used for categorical variables. Statistical assessments were two-tailed, and *p*<0.05 was considered significant. All statistical analyses were carried out using IBM SPSS statistical software version 24 for Windows (IBM Corp., Armonk, New York, USA).

## Results

### Characteristics of patients who underwent transsphenoidal operation

Clinical characteristics of the included patients are summarized in **Table 1.** No significant differences were found between patients in the one-and-half nostril operation group and the mono-nostril group in sex distribution, prior operation, and percentage with apoplexy (all *p*>0.05). The major tumor type in the two groups was non-functional tumor, with no significant difference between the two groups in tumor type (*p*>0.05). No significant differences were found between patients receiving a mononostril or one-and-half nostril operation in tumors with Knosp grade, Hardy suprasellar extension and preoperative tumor volume (all *p*>0.05).

**Table 1:** Demographic and clinical characteristics of patients undergoing endoscopic transsphenoidal operation for large pituitary tumors.

	Mono-nostril (n = 30)	One-and-half nostril (n = 48)	*P* value
**Age, mean (SD)**	47.13 ± 15.32	51.77 ± 16.03	0.194
**Gender**			0.816
Male, n (%)	14 (47%)	25 (52%)	
Female, n (%)	16 (53%)	23 (48%)	
**Prior operation, n (%)**	7 (23%)	9 (19%)	0.774
**Tumor type, n (%)**			0.213
Non-functional	24 (80%)	34 (71%)	
Prolactin	5 (17%)	5 (10%)	
Growth hormone	1 (3%)	8 (17%)	
Cushing	0 (0%)	1 (2%)	
Thyroid stimulating hormone	0 (0%)	0 (0%)	
**Apoplexy, n (%)**	13 (43%)	15 (31%)	0.335
**Knosp grade, n (%)**			0.337
0	1 (3%)	0 (0%)	
1	7 (23%)	7 (15%)	
2	9 (30%)	12 (25%)	
3	7 (23%)	20 (42%)	
4	6 (20%)	9 (19%)	
**Hardy suprasellar extension, n (%)**			0.065
A	7 (23%)	7 (15%)	
B	17 (57%)	16 (33%)	
C	4 (13%)	21 (44%)	
D	2 (7%)	4 (8%)	
**Preoperative tumor volume (cm**^**3**^**), mean (SD)**	6.10 ± 11.48	8.01 ± 8.87	0.136

SD, standard deviation.

*p<0.05.

### Surgical outcomes for pituitary tumors

**Table 2** lists the postoperative outcomes and complications of the two procedures. The operative time, hospital stay, and postoperative MRI extent of resection were not significantly different between patients receiving the mono-nostril approach and those receiving the one-and-half nostril approach (all *p*>0.05). After three months follow-up, significantly more patients who received the one-and-half nostril operation had a lower proportion of postoperative hypopituitarism (*p =* 0.016) than did those who received the mono-nostril operation. However, the rates of diabetes insipidus, improved visual field and complete or partial remission of endocrine function were similar in the two groups (all *p*>0.05). The rates of intraoperative CSF leakage, postoperative leakage, and re-operation for CSF leakage were also similar between the two operations. Also, the rates of surgical complications (contained intracranial infection, internal carotid artery injury, and cranial nerve palsy), nasal complications (contained sinusitis, epistaxis, hyposmia), and re-operation for nasal complications were similar in the two patient groups.

**Table 2:** Postoperative outcomes and complications of patients with large tumor receiving transsphenoidal operation using mono-nostril or one-and-half nostril approaches.

	Mono-nostril (n = 30)	One-and-half nostril (n = 48)	*p*-value
**Operation time (min, median, range)**	150 (47–600)	145.5 (65–302)	0.723
**Hospital stay (days, median, range)**	7 (3–90)	6.5 (3–68)	0.550
**Postoperative MRI extent of resection, n (%)**			0.810
Total (100%)	38 (60%)	31 (65%)	
Subtotal (<100%)	12 (40%)	17 (35%)	
**Visual field change, n (%)**			
Improved	9 (30%)	19 (40%)	0.471
Nil	21 (70%)	29 (60%)	
**Diabetes insipidus, n (%)**	6 (20%)	7 (15%)	0.548
**Hypopituitarism, n (%)**	7 (23%)	1 (2%)	0.004*
**Intraoperative CSF leakage, n (%)**	13 (43%)	16 (33%)	0.471
**Postoperative CSF leakage, n (%)**	3 (10%)	3 (6%)	0.670
**Surgical complication, n (%)**	4 (13%)	3 (6%)	0.419
Intracranial hemorrhage	1 (3%)	1 (2%)	
Infection	1 (3%)	1 (2%)	
Internal carotid artery injury	1 (3%)	0 (0%)	
Cranial nerve Palsy	1 (3%)	1 (2%)	
**Nasal complication, n (%)**	7 (24%)	14 (29%)	0.611
Sinusitis	5 (17%)	8 (17%)	
Epistaxis	2 (7%)	5 (10%)	
Hyposmia	0 (0%)	1 (2%)	
**Postoperative radiation therapy, n (%)**	4 (13%)	7 (15%)	1.000
**Postoperative endocrine function, n (%)**			0.521
Complete remission	5 (83%)	13 (93%)	
Partial remission	0 (0%)	1 (7%)	
Stable disease	1 (17%)	0 (0%)	
**Re-operation for CSF leakage, n (%)**	3 (10%)	3 (6%)	0.670
**Re-operation for nasal complication, n (%)**	6 (86%)	8 (57%)	0.337

MRI, magnetic resonance imaging; CSF, cerebrospinal fluid.

Selective assessment of surgical outcomes in all patients with small (diameter ≦1 cm) and large (diameter >1 cm) pituitary tumors receiving either of the two operations revealed that, as shown in **S1 Table**, patient characteristics were similar in the two groups (all *p* >0.05). Also, the surgical outcomes (**S2 Table**) were similar to those presented in **Table 2**, except that the difference in postoperative improvement in visual field change reached statistical significance (*p* = 0.044).

## Discussion

This is the first study to compare the efficacy and safety of endoscopic transsphenoidal surgery for treatment of pituitary macroadenoma using a single-surgeon with one-hand/mono-nostril or a two-hand/one-and-half nostril approach. However, previous study had revealed that an alternative to the two-surgeon approach is the use of a mechanical or pneumatic endoscope holder allowing a single-surgeon technique. Major findings of the present study were those of less frequent occurrence of hypopituitarism with the two-hand/one-and-half nostril approach than with the one-hand/mono-nostril approach. Differences between the operations in rates of postoperative hypopituitarism were noted for both small and large pituitary tumors, but the differences in visual changes reached statistical significance in this population. The rates of CSF leakage, surgical complications, and nasal complications were similar between the two operations. Overall, the findings support the safety and efficacy of the two-hand/one-and-half nostril operation for resection of large pituitary tumors.

Results in this series of operations by a single surgeon at a high-volume center are consistent with reports from several other centers [15, 27–29]. Those studies revealed that endoscopic transsphenoidal surgery can be carried out with low morbidity, short hospital stays, and an excellent extent of resection. High-volume centers employing a single surgeon have superior endocrine outcomes for patients with functional tumors than do lower-volume centers or those using multiple surgeons [30, 31].

Although the two-surgeon/four-hand method is the established method for performing these procedures, it also has disadvantages [32–37]. First, it requires two surgeons—one to operate the endoscope, and the other to free up the surgeon’s hands and use microsurgical techniques to remove the tumor. The frequent presence of two surgeons may limit neurosurgeons’ opportunity to become familiar with one-handed endoscopic surgery, intranasal exposure techniques, and nasal/paranasal anatomy, all of which require significant repetition to gain comfort. The main disadvantage is that the learning curve and depth of field of surgery may be problematic for some surgeons. Mamelak et al. [22] used a single-surgeon method, using a pneumatic endoscope stent as an alternative to the surgeon’s hand-held endoscope for all aspects of the operation beyond the initial nasal stage. Using this method, they performed many transsphenoidal surgeries, and the results were similar to those reported using the two-surgeon method, and in many cases were superior to these surgeries [22, 32–35, 37]. The present study expanded these findings and we used a single-surgeon method assisted with an endoscope holder to carry out two-hand/one-and-half nostril operation for pituitary macroadenoma. The advantages of all techniques employed in the two-surgeon endoscopic operation are incorporated into this approach. The results seem particularly good for functional or large pituitary tumor.

For transsphenoidal endoscopic surgery of pituitary tumors, single or double nostril methods can be used. The single nostril approach can limit surgical activity, but it also causes less trauma to the nose [36]. Wen et al. [36] have reported that patients undergoing double nostril surgery have transient diabetes insipidus, anterior pituitary insufficiency and shorter hospital stay, but epistaxis is more frequent than single nostril surgery. Wen et al. [36] also found that the bi-nostril approach allows greater instrument flexibility, while one-hand/single nostril surgery, such as the three-hand technique [13], may be challenging for intraoperative movement of the instrument. Regardless of the width and depth of entry, the wide panorama of the endoscope allows good visualization, supporting the use of a modified single nostril approach to achieve good visualization of the saddle area [13]. In this method, the surgeon places the endoscope at the top of the nostril, placing the endoscope at the level of the tip of the nose, and then introduces a long and thin suction underneath through the same nostril, with a small, low-key curette. With this arrangement, the surgeon can manipulate slowly but steadily while maintaining the flexibility of the instrument. This method requires more extensive coagulation and manipulation of the mucosa and turbinate, but it provides a larger working area in the sphenoid sinus [6]. Particularly, the present study further showed that single-surgeon two-hand/one-and-half nostril endonasal transsphenoidal operation for treatment of pituitary macroadenoma have lower frequent of hypopituitarism and shorter hospital stay. In addition, further studies should be conducted to determine whether a single-surgeon using this modified mono-nostril approach improves postoperative outcomes in patients with pituitary tumor.

As in previous studies [6, 8, 36], the present study found a similar extent of resection and complication rates between the mono-nostril and bi-nostril approaches. Still other studies [8, 38, 39], have reported more frequent total resections of cavernous sinus invasive tumors with the double nostril approach. In the present study, no significant differences were found in total operation time between patients who underwent one-hand/single nostril surgery (150 minutes) vs. the two-hand/one-and-a-half nostril surgery (145 minutes). Due to the numerous variables, it is impossible to confidently compare these times with the times reported in the literature, but Eseonu et al. [13] reported an operation time of 162 minutes, which is longer than our operation time in pituitary tumor surgery using the single nostril three-hand technique; it is also reported that the operation time in single nostril surgery is shorter [6, 8]. Other reports on the average operation time of endoscopic transsphenoidal cases range from 102 to 255 minutes [40–42].

Tumor-related hypopituitarism is another serious problem that needs to be addressed, mainly because postoperative hypopituitary hypofunction is associated with high overall mortality [43]. Recently, pooled data showed that patients with endoscopically treated non-functioning pituitary adenomas (NFPA) tend to have a lower percentage of postoperative pituitary dysfunction, with higher total resection rates and higher rates of visual acuity improvement [44]. Recovery of pre-existing hypopituitarism after transsphenoidal surgery for pituitary adenoma is an important research result. Little et al. characterized pituitary gland outcomes with a focus on gland recovery following endoscopic transsphenoidal removal of clinically nonfunctioning adenomas [45]. Complete endoscopic pituitary surgery improves the function of the pituitary gland in a very small number of patients, and the most likely recoverable defect is adrenal insufficiency [45]. However, no comparison has been made between the effects of one-handed/single nostril and two-handed/single-half nostril endoscopic transsphenoidal resection on hypopituitary dysfunction after pituitary tumor-related surgery. In the present study, we found a tendency towards better outcomes with the one-and-half nostril operation for invasive macroadenomas and more improvement in visual field change and less hypopituitarism, especially with macroadenomas. The dual nostril approach allows greater instrument flexibility, reduces transient diabetes insipidus and anterior pituitary insufficiency, and allows shorter hospital stays [36]. According to these findings, we have speculated that the difference in the frequency of hypopituitarism between the two techniques may be associated with instrument flexibility. Certainly, it merits further investigations.

The present study has several limitations. It is a retrospective study, which has the potential for biases. The study cohort was not large, and the study was conducted in a single medical center in Taiwan, which specializes in surgery for pituitary tumors; thus, our results may not be representative of those from other centers. The sample size did not allow us to reach definitive conclusions. Prospective, large, rigorously controlled studies are needed to reliably compare various surgical methods for endoscopic endonasal transsphenoidal resection of pituitary tumors.

## Conclusions

Our work supports that single-surgeon two-hand/one-and-half nostril endonasal transsphenoidal operation is effective and safe for resection of pituitary macroadenoma. This surgical approach may avoid the main disadvantage of two-surgeon/four-hand method, which have a better learning curve and depth of field of surgery.

## Supporting information

S1 Table Clinical characteristics of patients undergoing endoscopic transsphenoidal operation for small and large pituitary tumors. (DOCX)

S2 Table Postoperative outcomes and complications of patients operated with mono-nostril or one-and-half nostril approaches. (DOCX)
