# Supplementary material for: Endoscopic transsphenoidal surgery for resection of pituitary macroadenoma: A retrospective study
Source: PLoS One. 2021 Aug 6;16(8):e0255599. doi: 10.1371/journal.pone.0255599 (PMC8345891; doi:10.1371/journal.pone.0255599)
Supplement: S1 Table — (DOCX) [file pone.0255599.s001.docx]

| **S1 Table. Clinical characteristics of patients undergoing endoscopic transsphenoidal operation for small and large pituitary tumors** | | | |
| --- | --- | --- | --- |
|  | **Mono-nostril**  **(n = 50)** | **One-and-half nostril (n = 50)** | ***P* value** |
| **Age, mean (SD)** | 44.94 ± 14.48 | 51.48 ± 15.78 | 0.032* |
| **Gender** |  |  | 0.225 |
| Male, n (%) | 18 (36%) | 25 (50%) |  |
| Female, n (%) | 32 (64%) | 25 (50%) |  |
| **Prior operation, n (%)** | 8 (16%) | 9 (18%) | 1.000 |
| **Apoplexy, n (%)** | 14 (28%) | 16 (32%) | 0.828 |
| **Tumor type, n (%)** |  |  | 0.505 |
| Non-functional | 33 (66%) | 34 (68%) |  |
| Prolactin | 8 (16%) | 6 (12%) |  |
| Growth Hormone | 4 (8%) | 8 (16%) |  |
| Cushing | 3 (6%) | 2 (4%) |  |
| Thyroid stimulating hormone | 2 (4%) | 0 (0%) |  |
| **Tumor size, n (%)** |  |  | <0.001* |
| Microadenoma (<1 cm) | 18 (36%) | 2 (4%) |  |
| Macroadenoma (1-3 cm) | 25 (50%) | 26 (52%) |  |
| Giant tumor (>3 cm) | 7 (14%) | 22 (44%) |  |
| **Knosp grade, n (%)** |  |  | < 0.001* |
| 0 | 12 (24%) | 0 (0%) |  |
| 1 | 15 (30%) | 9 (18%) |  |
| 2 | 9 (18%) | 12 (24%) |  |
| 3 | 8 (16%) | 20 (40%) |  |
| 4 | 6 (12%) | 9 (18%) |  |
| **Hardy suprasellar extension, n (%)** |  |  | <0.001* |
| A | 26 (52%) | 9 (18%) |  |
| B | 18 (36%) | 16 (32%) |  |
| C | 4 (8%) | 21 (42%) |  |
| D | 2 (4%) | 4 (8%) |  |
| **Preoperative tumor volume (cm^3^), mean (SD)** | 3.73 ± 9.31 | 12.88 ± 3.81 | <0.001* |
| SD, standard deviation. **p*<0.05 | | | |
