# Supplementary material for: Endoscopic transsphenoidal surgery for resection of pituitary macroadenoma: A retrospective study
Source: PLoS One. 2021 Aug 6;16(8):e0255599. doi: 10.1371/journal.pone.0255599 (PMC8345891; doi:10.1371/journal.pone.0255599)
Supplement: S2 Table — (DOCX) [file pone.0255599.s002.docx]

| **S2 Table. Postoperative outcomes and complications of patients operated with mono-nostril or one-and-half nostril approaches** | | | |
| --- | --- | --- | --- |
|  | **Mono-nostril**  **(n = 50)** | **One-and-half nostril**  **(n = 50)** | ***p*-value** |
| **Operation time (mins, median, range)** | 121.5 (47- 600) | 144.5 (65-302) | 0.202 |
| **Hospital stay (days, median, range)** | 6 (3-90) | 6.5 (3-68) | 0.210 |
| **Postoperative MRI extent of resection, n (%)** |  |  | 0.378 |
| Total (100%) | 38 (76%) | 33 (66%) |  |
| Subtotal (<100%) | 12 (24%) | 17 (34%) |  |
| **Residual of large tumor, n (%)** |  |  |  |
| Diameter≥ 3 cm | 5/7 (71.4%) | 12/22 (54.5%) | 0.665 |
| Knosp grade ≥3 | 10/14 (71.4%) | 16/32 (50%) | 0.212 |
| Hardy score ≥C | 5/6 (83.3%) | 10/25 (40%) | 0.072 |
| **Visual field change, n (%)** |  |  |  |
| Improved | 9 (18%) | 19 (38%) | 0.044* |
| Nil | 41 (82%) | 31 (62%) |  |
| **Diabetes insipidus, n (%)** | 9 (18%) | 7 (14%) | 0.786 |
| **Hypopituitarism, n (%)** | 9 (18%) | 1 (2%) | 0.016* |
| **Intraoperative CSF leakage, n (%)** | 15 (30%) | 16 (32%) | 1.000 |
| **Postoperative CSF leakage, n (%)** | 6 (12%) | 3 (6%) | 0.487 |
| **Surgical complication, n (%)** | 4 (8%) | 3 (6%) | 1.000 |
| Intracranial hemorrhage | 1 (2%) | 1 (2%) |  |
| Infection | 1 (2%) | 1 (2%) |  |
| Internal carotid artery injury | 1 (2%) | 0 (0%) |  |
| Cranial nerve palsy | 1 (2%) | 1 (2%) |  |
| **Nasal complication, n (%)** | 12 (24%) | 15 (30%) | 0.653 |
| Sinusitis | 9 (18%) | 8 (16%) |  |
| Epistaxis | 2 (4%) | 6 (12%) |  |
| Hyposmia | 1 (2%) | 1 (2%) |  |
| **Postoperative radiation therapy, n (%)** | 4 (8%) | 7 (14%) | 0.338 |
| **Postoperative endocrine function, n (%)** |  |  | 1.000 |
| Complete remission | 14 (88%) | 14 (93%) |  |
| Partial remission | 1 (6%) | 1 (7%) |  |
| Stable disease | 1 (6%) | 0 (0%) |  |
| **Re-operation for CSF leakage, n (%)** | 6 (12%) | 3 (6%) | 0.487 |
| **Re-operation for nasal complication, n (%)** | 9 (75%) | 9 (60%) | 0.683 |
| MRI, magnetic resonance imaging; CSF, cerebrospinal fluid. **p*<0.05 | | | |
